# Fracture strength of direct occlusal veneers with different short fiber-reinforced composite cores and veneering materials: an in-vitro study

**DOI:** 10.1007/s00784-024-06013-6

**Published:** 2024-11-11

**Authors:** Menna Moataz Aboelnor, Khaled Aly Nour, Heba Mohamed Ahmed Al-Sanafawy

**Affiliations:** 1 Department of Operative Dentistry, Faculty of Dentistry, Ain Shams University, Cairo, Egypt; 2 Department of Conservative Dentistry, School of Dentistry, New Giza University, Giza, Egypt

**Keywords:** Fiber-reinforced composite resin, Direct occlusal veneers, Injection molding, Occlusal replica technique, Bi-structured restorations, Injectable composite resin

## Abstract

**Objective:**

The objective of this study is to evaluate the effectiveness of two different viscosities of short fiber-reinforced composite resin (SFRC) cores and two different viscosities of veneering composite resins on the fracture strength of direct occlusal veneers.

**Materials and methods:**

One maxillary premolar was virtually prepared for an occlusal veneer and printed into resin dies. In total, (*n* = 48) resin dies were printed and assigned into three groups according to the type of core material of the occlusal veneer; mono-structured without a SFRC core, a high viscosity SFRC core, and a low viscosity SFRC core. Each group was re-divided into two subgroups (*n* = 8) according to the veneering composite resin; packable composite resin and injectable composite resin. Mono-structured and bi-structured direct occlusal veneers were fabricated on the resin dies using the mentioned core and veneering materials with the occlusal replica technique. Fracture strength was evaluated using a universal testing machine and the mode of failure was inspected. Statistical analysis to compare the core and veneering materials was performed using independent t test (*P* ≤ 0.05) and one-way ANOVA followed by tukey’s post hoc test (Pa ≤ 0.0166) when appropriate. Interactions between subgroups were tested using two-way ANOVA, and one-way ANOVA was used to compare all subgroups followed by tukey’s post hoc test (Pa ≤ 0.0033). Intergroup comparison between failure modes were performed using chi square test (Pa ≤ 0.0033).

**Results:**

The presence of a SFRC core significantly improved the fracture strength of the specimens. There was no significant difference between the fracture strength of high viscosity SFRC and low viscosity SFRC cores. Specimens veneered with injectable composite resin had significantly superior fracture strength compared to packable composite resin. Additionally, there was a weak correlation between fracture strength and mode of failure.

**Conclusion:**

Short fiber reinforced composite resin significantly increases the fracture strength of direct occlusal veneers. Injectable composite resin has significantly higher fracture strength than packable composite resin as veneering materials of direct occlusal veneers.

**Clinical relevance:**

Bi-structured direct occlusal veneers fabricated of injectable composite resin with low viscosity SFRC cores can withstand high masticatory forces in stress-bearing areas.

## Introduction

Occlusal veneers are restorations that restore the occlusal surface without axial wall involvement [1]. These restorations are mainly indicated in cases of lost form and function of the occlusal surface including tooth wear [2], amelogenesis imperfecta [3], and molar-incisor-hypo mineralization [4]. 

Directly fabricated occlusal veneers represent a conservative approach with many advantages including providing single visit restorations, being cost-effective, requiring a more conservative cavity preparation in comparison to indirect occlusal veneers, easy repairability, in addition to having resiliency close to that of natural dental tissue [5, 6]. However, these restorations require a great skill from the operator to assure good margins and occlusal anatomy [6]. This problem can be addressed by the occlusal replica technique, a direct or semidirect method that transfers an extra-oral model of the restoration into a transparent negative replica, into which the restorative material is placed and then adapted on the tooth for accurate occlusal reproduction [7]. Additionally, direct restorations frequently require repairs for bulk-fracture [5], as it one of the most common causes of failure seen in composite resin restorations [8]. This can be encountered by adding short fiber-reinforced composite (SFRC) as a dentine-replacing substructure of the direct restoration. As short fiber-reinforced composite resin (SFRC) allows better resistance to crack initiation and propagation when compared to particulate-filled composite resin (PFC), either through crack deflection, delaying, or ceasing [9]. Accordingly, the rationale behind bi-structured or bi-layered restorations with a SFRC core as a substructure or as a dentine-replacing material veneered with PFC is to improve the mechanical properties of the restored tooth in terms of fracture strength [5]. 

The effect of SFRC cores has been previously studied on bi-structured directly restored mesio-occluso-distal cavities [10], endodontically treated teeth [11] as well as onlays [12]. However, reviewing the current literature revealed the need to study its effect on occlusal veneers. Hence, this study aimed to investigate the effect of SFRC on the fracture strength of occlusal veneers, in addition to investigating the effect of different viscosities of SFRC cores ( high viscosity: EverX Posterior, GC, Japan; low viscosity: EverX Flow, GC, Japan) on fracture strength as well as different viscosities of veneering materials ( packable composite resin: G-aenial A’CHORD, GC Europe, Belgium; injectable composite resin: G-aenial Injectable, GC, Japan).

The first null hypothesis of this study was that SFRC will not affect the fracture strength of occlusal veneers. The second hypothesis was that the viscosity of the SFRC core material will have no effect on fracture strength. The third hypothesis was that the viscosity of the veneering material of bi-structured restorations will have no effect on fracture strength.

## Materials and methods

### Specimen preparation

**Fig. 1:**
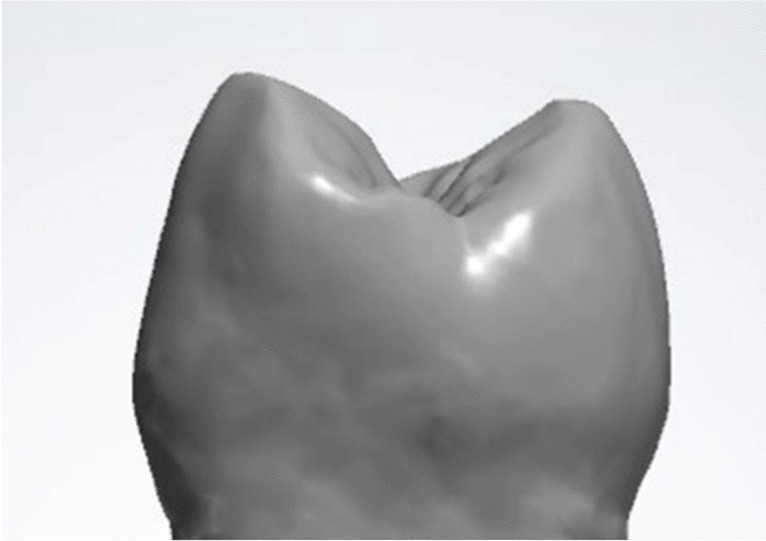
Pre-operative scan of a maxillary premolar using a digital intraoral scanner (CEREC Omnicam, Dentsply Sirona, USA)

A maxillary human premolar was scanned as a pre-operative biocopy using a digital intraoral scanner (CEREC Omnicam, Dentsply Sirona, USA) **(**Fig. 1**)** [13]. The tooth was virtually prepared for an occlusal veneer using computer aided digital dental design software (Exocad Dental CAD, Exocad GmbH, Germany) [1]. This preparation resulted in an anatomical occlusal reduction with butt joint margins.

**Fig. 2:**
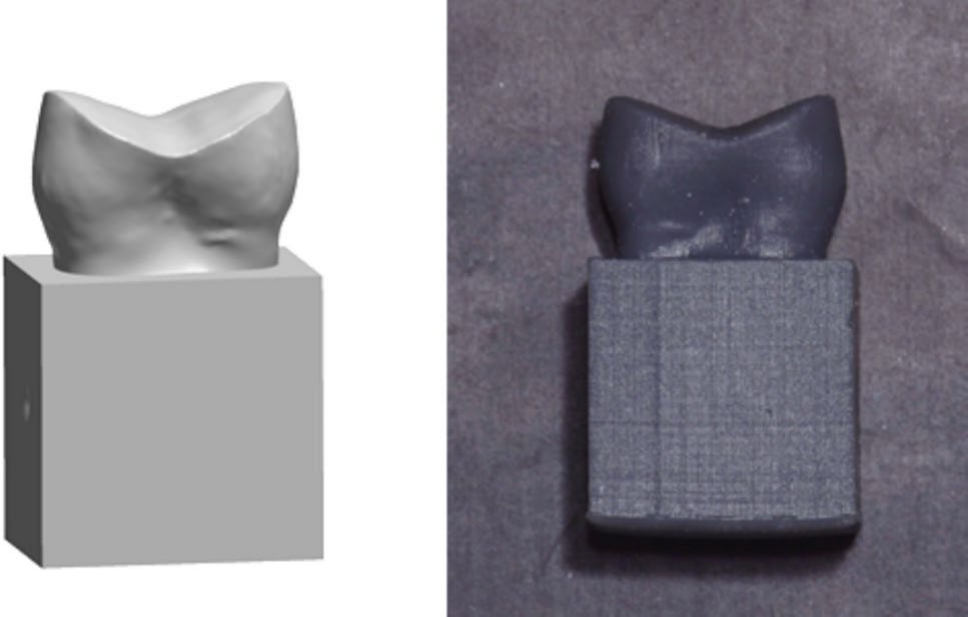
STL.files of the scanned prepared tooth and the cuboidal base were merged and printed into resin duplicate dies (Tough 1500 Resin, Formlabs, Inc., USA)

To ensure a proper fit with the seating area of the universal testing machine (Instron 3365, Instron industrial products, USA), a cuboidal base was designed and merged into the virtually prepared tooth. This three-dimensional model was then printed into duplicate dies using grey printing resin (Tough 1500 Resin, Formlabs, Inc., USA) by a 3D stereolithography printer (Form 2, Formlabs, Inc., USA) [14]. The printing parameters were set to a layer thickness of 100-microns and a laser spot size of 140 microns. After printing, the material was post-cured for 60 minutes at 70 °C using a post-processing unit (Form Cure, Formlabs, Inc., USA) **(**Fig. 2**)**.

Sample size calculation was performed using G*Power version 3.1.9.7, setting the power at 80%. The predicted total sample size (n) was found to be (48) samples, making it (n=16) for each group & (n=8) for each subgroup [15]. Accordingly, a total of 48 resin dies were printed in this study for occlusal veneer fabrication**. **(Table 1) summarizes the materials used in this study for restoration fabrication. Resin dies were divided into 3 groups (n=16) according to the type of core used; the first group was mono-structured without SFRC reinforcement, the second group had a high viscosity SFRC core (EverX Posterior, GC, Tokyo, Japan), and the third group had a low viscosity SFRC core. (EverX Flow, GC, Tokyo, Japan).  Each group was further divided according to the veneering composite resin used to two equal subgroups (n=8); a packable composite resin (G-aenial A’CHORD, GC Europe, Belgium) subgroup and an injectable composite resin (G-aenial Injectable, GC, Tokyo, Japan) subgroup. (Table 2)  presents a summary of the study design

**Table 1:** Material description, composition, manufacturer and lot number

Material	Description	Composition	Manufacturer	Lot number
Beautibond Universal Adhesive	Universal dental adhesive	Phosphonic acid monomer, carboxylic acid monomer, Bis-GMA, TEG-DMA, acetone, water, initiators	Shofu, Kyoto, Japan	2002182
G-aenial A’CHORD, Shade A3	Packable nanohybrid composite resin	Bis-MEPP, Bis-EMA, TEGDMA, UDMA glass-filler, barium glass, fumed silica. Filler loading: 81–82 wt%	GC Europe, Belgium	2103041
G-aeinal Injectable, Shade A3	Injectable nanohybrid composite resin	UDMA, Bis-EMA, dimethacrylate monomers barium glass, silica; photoinitiator. Filler loading: 69 wt%	GC, Tokyo, Japan	2202241
EverX Flow, Bulk shade	Low-viscosity short fiber-reinforced composite resin	Bis-EMA, TEGDMA, UDMA, Short E- glass fiber, barium glass .Fiber loading: 70 wt%	GC, Tokyo, Japan	2112021
EverX Posterior	High-viscosity short fiber-reinforced composite resin	Bis-GMA, TEGDMA, PMMA, Short E-glass fiber, barium glass and silicon dioxide. Fiber loading: 74.2 wt%	GC, Tokyo, Japan	2108253

*Bis-MEPP,* Bisphenol a ethoxylate dimethacrylate; *Bis-EMA,* Bisphenol A ethoxylated dimethacrylate; *TEGDMA,* Triethylene glycol di-methacrylate; *UDMA,* Diurethane dimethacrylate; *PMMA,* Poly-methyl methacrylate

**Table 2:** Experimental design (*n* = 8)

Core Material	Veneering Material	Group (*n* = 8)
Mono-structured: No SFRC	Packable composite resin	NP
	Injectable composite resin	NI
High viscosity SFRC	Packable composite resin	HP
	Injectable composite resin	HI
Low viscosity SFRC	Packable composite resin	LP
	Injectable composite resin	LI

**Fig. 3:**
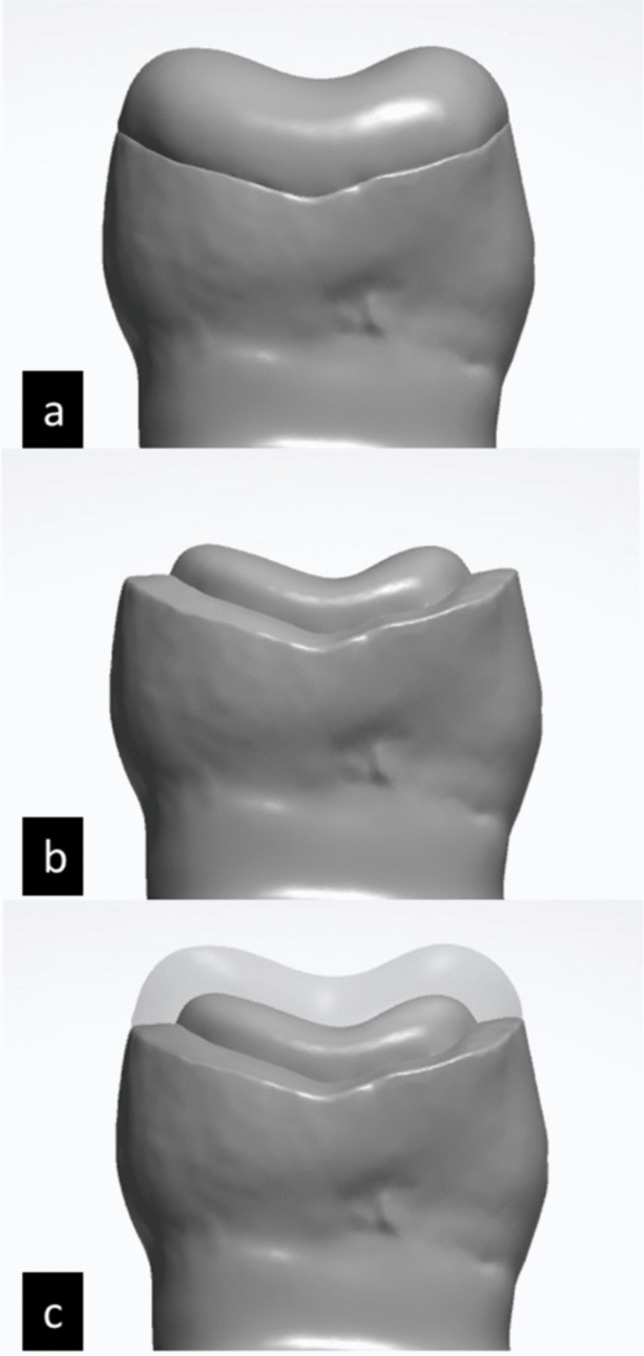
Computer Aided Designing of the occlusal veneer** a **Occlusal veneer design **b** Core design  **c** End result; a bi-structured occlusal veneer

 The occlusal veneer was designed in two stages using the same dental design software. The resultant design was a bi-structured occlusal veneer of an even 2-mm thickness, composed of two layers; a 1-mm core and a 1-mm veneering layer [1, 15]. The first stage was designing the occlusal veneer with an even 2-mm occlusal height guided by the pre-operative scan. In second stage, the core of the occlusal veneer was designed by performing a 1-mm cutback of the restoration circumferentially and occlusally, creating a core for the SFRC [13, 16] (Fig. 3).

**Fig. 4:**
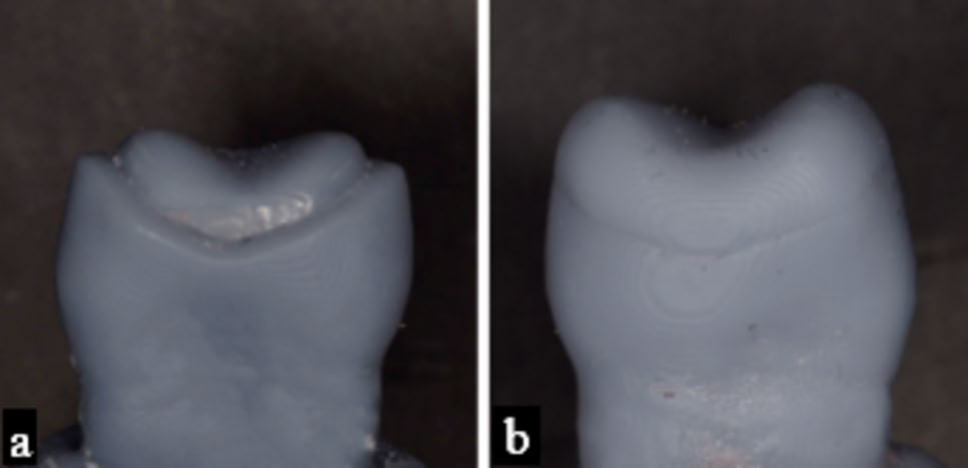
Two models were printed into grey resin (Grey Resin V5, Formlabs, Inc., USA) for fabrication of clear indices (**a**) model of the designed core (**b**) model of the designed veneer

After completing the design process, two models were printed into grey resin (Grey Resin V5, Formlabs, Inc., USA); one of the designed core and one of the designed occlusal veneer (Fig. 4). For standardization, clear addition silicon (Exaclear, GC Europe) was used to record each model, creating clear indices for restoration fabrication [7]. 

**Fig. 5:**
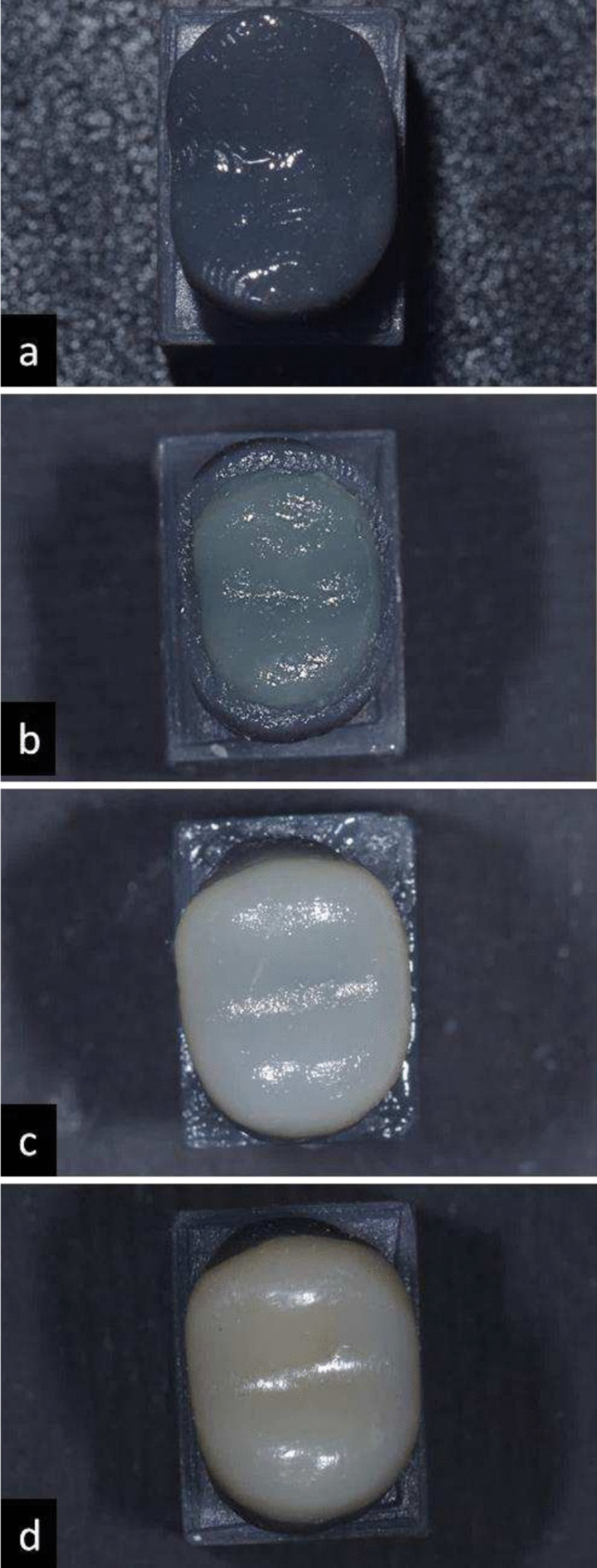
Direct occlusal veneer fabrication using transparent indices  **a** adhesive preparation  **b** SFRC core fabrication in bi-structured groups **c** occlusal veneer fabrication **d** after polishing

Prior to restoration fabrication, a universal dental adhesive was applied to each resin die following the manufacturer’s instructions (BeautiBond Universal Adhesive, Shofu, Kyoto, Japan) and light polymerized using a light curing unit (Elipar™ DeepCure-L LED, 3 M, Germany) directed perpendicularly on the adhesive (Fig. 5a). [17] The clear indices were then used for the fabrication of the direct occlusal veneers by the occlusal replica technique on the resin dies with the materials listed in **(**Table 1**)** according to their assigned group as proposed in **(**Table 2**)**.

For subgroups receiving a high viscosity SFRC core and those veneered with packable composite resin, the material was packed inside the clear index then pressed on the resin die till full seating. For subgroups receiving a low viscosity SFRC core and those veneered with injectable composite resin, the material was injected through the clear index using a small hole made above the buccal cusp tip as a channel; this hole was made using the injection tip provided with the mentioned materials. Each index was used for 8 specimens then discarded to avoid distortion. Each core and veneer was light cured using a light curing unit (Elipar™ DeepCure-L LED, 3 M, Germany) for 20 s directed perpendicular to the surface, then re-cured for 20 s after removing the index [10, 18] (Fig. 5b, c ).

Excess composite resin on each specimen was scraped away and removed using a No.15 blade. Then polished using a one-step polishing system (Enhance Polishing Cup, Dentsply Sirona, Germany), by applying three gentle strokes on each aspect of the restoration, noting that each cup was used for 8 specimens then discarded. (Fig. 5d) [19] Finally, each specimen was inspected using 4x magnification loupes (Univet, Univet Loupes Spa, Italy) with an attached headlight (EOS 2.0, Univet, Italy) to ensure the absence of any internal or external defects, voids, irregularities, open margins, and marginal inadaptation between each resin die and occlusal veneer.

### Fracture strength test

**Fig. 6:**
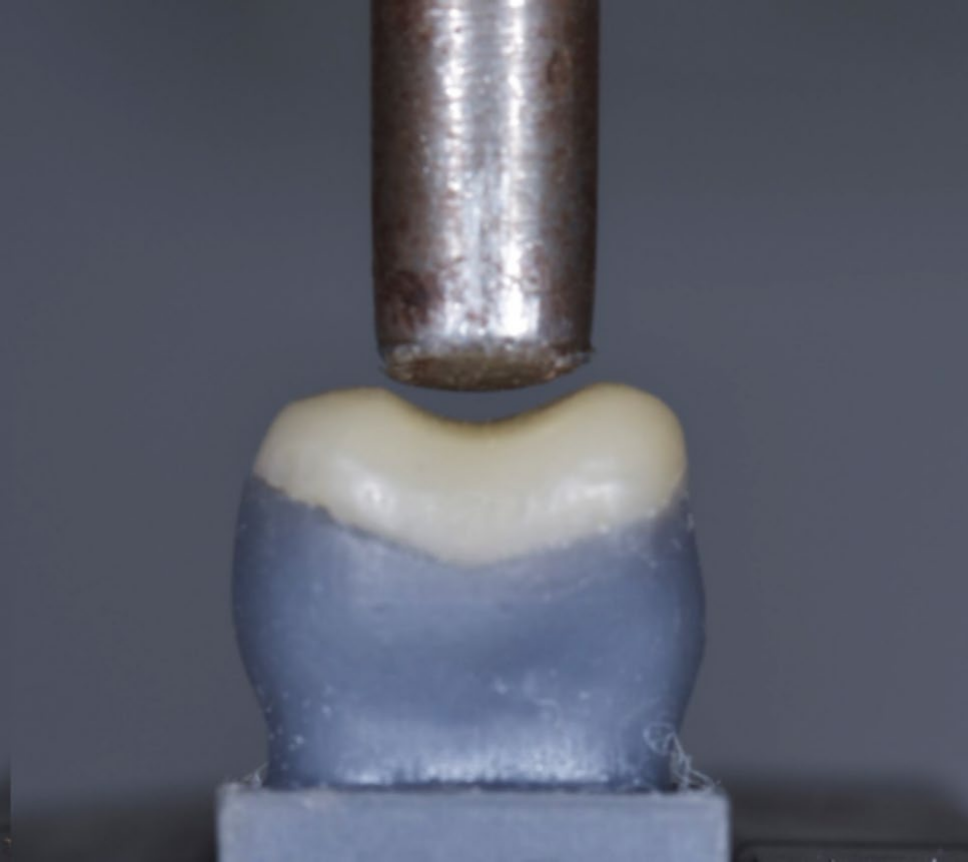
Specimens were installed in fixed compartment of universal testing machine (Instron, 3365, Instron industrial products, USA) along with a 6mm cylindrical attachment at the upper compartment allowing a 2-point-contact

 Testing fracture strength was performed using a universal testing machine (Instron 3365, Instron industrial products, USA) [20] (Fig. 6). In the lower compartment of the machine each resin die was firmly seated using tightening screws. In the upper compartment of the machine a cylindrical attachment of 6 mm was centered over the specimen for axial loading. This cylindrical attachment was selected to touch each occlusal veneer at the buccal and palatal cusp inclines, ensuring a 2-point-contact with each specimen [21]. Occlusal axial loading was applied on each specimen at a 90-degree angle, at a rate of 1 mm/minute and till maximum loading at 5000 N (N), fracture strength was detected visually, audibly and observed as a drop at the load-deflection curve [21–23].

Initial failure load (IF) in Newton (N) was recoded for each specimen along with the load-deflection curve of each subgroup using computer software (Instron BlueHill Universal computer software) [23, 24]. Additionally, the mode of failure for each specimen was visually examined and documented. Failures were either identified as favorable failures that can be repaired (cracks within the restoration/cracks extending from the restoration till parts of the cusps/ separated broken segments of the restoration), or unfavorable failures that can’t be repaired (fractures extending till the neck of the resin die/ fractures splitting the crown/ fractures causing complete separation between the crown and the base of the resin die) [22].

### Data analysis

Statistical power of the study was set at 80% with a 95% confidence level. Data was explored for normality using Kolmogrov Smirnov test and Shapiro Wilk test. Continuous data of the veneer and core materials showed normal distribution and were described using mean and standard deviation. Intergroup comparison between continuous variables of the veneer and core materials was performed using independent t test (*P* ≤ 0.05) and one-way ANOVA followed by tukey’s post hoc test (P^a^ ≤ 0.0166) when appropriate. Cohen’s d was used to calculate effect sizes. Categorical data of the mode of failure was described as frequency and percentage. Intergroup comparison between categorical data was performed using chi square test (P^a^ ≤ 0.0033). Two-way ANOVA was used to test interaction of variables, one-way ANOVA was used to compare all subgroups followed by Tukey’s post hoc test (P^a^ ≤ 0.0033) and all tests were two tailed. Correlation between fracture strength values and failure mode was assessed using spearman’s rank correlation.

**Fig. 7:**
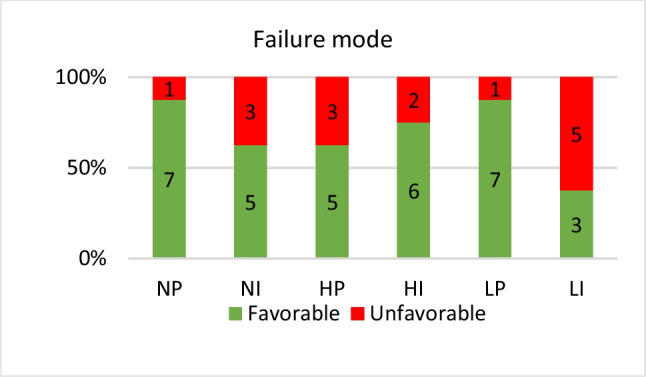
Bar chart showing frequency and percentage of failure mode within each subgroup

## Results

To study the effect of SFRC core materials, intergroup comparison within packable composite resin veneers revealed a statistically significant difference between the mono-structured subgroup (NP) and the bi-structured subgroups (HP and LP) (*P* < 0.001). NP demonstrated the least fracture strength, while HP and LP demonstrated nearly similar fracture strength. Similarly, intergroup comparison within injectable composite resin veneers revealed statistically significant difference between NI, HI and LI (*P* < 0.001). NI demonstrated the least fracture strength, while HI and LI demonstrated nearly similar fracture strength.

The type of SFRC core, whether high viscosity (HP) or low viscosity SFRC (LP), did not demonstrate a statistically significant difference within packable composite resin veneers (*P* = 0.9947). Cohen’s d effect size was insignificant (0.003). Which was a similar finding within injectable composite resin veneers, as subgroups with high viscosity and low viscosity SFRC cores ( HI and LI) did not demonstrate a significant difference (*P* = 0.3689). Cohen’s d effect size was small (0.46).

The type of veneering material, whether packable composite resin or injectable composite resin, revealed a statistically significant difference at intergroup comparison within mono-structured veneers (NI and NP), NI demonstrated higher fracture strength than NP (*P* = 0.0002). Cohen’s d effect size was very large (2.44). Similarly, in intergroup comparison within bi-structured veneers with high viscosity SFRC cores (HP and HI), HI demonstrated higher fracture strength than HP (*P* = 0.0357). Cohen’s d effect size was very large (1.16). And the same was observed in intergroup comparison within bi-structured veneers with low viscosity SFRC cores (LP and LI), as LI demonstrated higher fracture strength than LP (*P* = 0.0026). Cohen’s d effect size was very large (1.82).

To study the effect of interaction of veneer and core material on fracture strength, One-way ANOVA revealed statistically significant difference between subgroups (*P* < 0.001) (Table 3).

**Table 3:** Grouping information of fracture strength using the Tukey method and 95% confidence

Group	Mean	SD
NP	1003.32^a^	115.36
HP	1527.82^bc^	147.54
LP	1527.17^bc^	226.58
NI	1348.21^b^	163.15
HI	1771.10^cd^	256.68
LI	1865.85^d^	131.96
P value	*P* < 0.001	

Two-way ANOVA as shown in (Table 4) revealed a statistically significant effect of veneer material and core on fracture strength (*P* < 0.001), while interaction of veneer material and core had no statistically significant effect (*P* = 0.675). LI demonstrated the highest statistically significant fracture strength, whereas NP demonstrated the least. Intergroup comparison between failure modes was performed using chi square test (Pa ≤ 0.0033). Failure mode analysis revealed a weak correlation between fracture strength and failure mode (rho = 0.222), (*P* = 0.1290). Additionally, there was no statistically significant difference between subgroups in failure mode (*P* = 0.2447). The main failure mode in the current study was favorable, observed in 68.7% of the specimens. Among the mono-structured occlusal veneers, 75% showed favorable failures, while 65.6% of the bi-structured occlusal veneers with an SFRC core exhibited favorable failures (Fig. 7).

**Table 4:** Two-way ANOVA showing interaction of veneer and core material on fracture strength

Source	Sum of Squares	DF	Mean Square	F	P
Veneer (packable or injectable)	1145380.700	1	1145380.700	35.002	< 0.001
SFRC Core (without or high viscosity or low viscosity)	2654751.836	2	1327375.918	40.564	< 0.001
Veneer*Core	25949.313	2	12974.657	0.396	0.675
Residual	1374366.431	42	32723.010		

## Discussion

This in-vitro study aimed to investigate the effect of SFRC cores on the fracture strength of direct occlusal veneers, as well as the effectiveness of different viscosities of short fiber-reinforced composite (EverX Flow and EverX Posterior) and different viscosities of veneering composite resin (G-aenial A’CHORD and G-aenial Injectable) on fracture strength. In order to identify the benefit of this bi-structured design, particularly for occlusal veneers in areas subjected to high forces [13]. 

To ensure standardization among specimens, resin duplicates were used (Tough 1500 Resin, Formlabs, Inc., USA) to minimize dimensional discrepancies between specimens. Even though the resin duplicates don’t resemble the complex human dentition and are considered weaker than the natural tooth, their use was necessary to ensure standardization of all samples and elimination of human variations as proposed by Elsayed et al., [14] and Zimmermann et al., [25]. Additionally, transparent silicone indices using clear PVS (Exaclear, GC Europe) were used for restoration fabrication as described by Mehta et al., [7]. For an optimal design, the bi-structured occlusal veneer included a 1-mm veneering layer as according to Lassila et al., [26] the optimum thickness of the veneering composite resin of bi-structural restorations should range between 0.5 mm and 1 mm. To avoid stress concentration, only the basic anatomy of the occlusal surface of a maxillary premolar was designed, no supplemental grooves were featured. Fracture strength was tested through single loading using a universal testing machine (Instron 3365, Instron industrial products, USA) [20]. Even though cyclic loading simulates the clinical situation more than single loading [27], it was essential to first determine the fracture strength of bi-structured occlusal veneers, to assess their ability to withstand the masticatory occlusal load on premolars before conducting cyclic loading or aging tests, as they are considered time-consuming fatigue tests [27]. 

According to the results of this study, the presence of a SFRC core significantly increased the fracture strength of occlusal veneers, whether the veneering material was packable composite resin or injectable composite resin. Hence, the first null hypothesis that SFRC will not affect fracture strength was rejected. This is in harmony with the findings of Garoushi et al. [28], Barreto et al. [29], and Magne et al., [10] as their studies demonstrated a significant improvement in fracture strength for groups with SFRC cores in comparison to their control groups. The increase in fracture strength attributed to SFRC may have been due to the support provided by the randomly oriented fibers forming the core [28], which allow force dissipation throughout the restoration, increasing the load-bearing [29]. 

The second null hypothesis of this study was that the viscosity of the SFRC will have no effect on fracture strength was accepted. Subgroups having a high viscosity SFRC core and subgroups having a low viscosity SFRC core exhibited nearly similar fracture strength. This is in agreement with Agrawal et al., [30] as their study noted that the effect of the fiber-reinforced core is mainly influenced by the orientation of fibers rather than the viscosity. Fibers reinforce the restoration when oriented perpendicular to the long axis of applied forces [30], which is present in the randomly oriented multi-directional fibers of the SFRC used in this study, regardless the viscosity.

The third null hypothesis was that the viscosity of the veneering material will have no effect on fracture strength. This hypothesis was rejected. The type of veneering composite resin showed a significant effect on the fracture strength of occlusal veneers. Subgroups veneered with injectable composite resin demonstrated superior fracture strength compared to subgroups veneered with packable composite resin, whether mono-structured or bi-structured. However, Fráter et al., [27] in their study found no significant difference between the veneering materials, which may be attributed to the type of loading used; their specimens were subjected to cyclic loading until fracture, while this study employed single loading until initial fracture.

The interaction between veneer and core materials did not have a significant effect. The bi-structured subgroup fabricated of injectable composite resin as the veneering material and low viscosity SFRC as the core material demonstrated the highest fracture strength with a mean value of approximately 1866 N. This may be attributed to the resiliency of the materials forming this bi-structured occlusal veneer, which facilitates better force dissipation, and reduces stress concentration and crack formation. Additionally, this may have been a result of the materials’ ability to flow, allowing better adaption and less void formation, whether between the resin die and the restoration or between the veneer and the SFRC core [31]. The mono-structured subgroup fabricated of packable composite resin showed the least fracture strength with a mean value of approximately 1003 N. This may be due to the less resistance of fillers in this type of PFC to crack propagation compared to fibers, in addition to its high viscosity, which may result in microvoid formation or incomplete adaptation between the resin die and restoration. However, the average bite load on premolars typically ranges between 220 and 450 N [32], with the maximum bite load on premolars reaching 800 N [33], which remains significantly less than the fracture strength of this subgroup.

Failure analysis was visually identified for each specimen as described by Chang et al., [24] to identify whether the failure was favorable (repairable) or unfavorable (unrepairable) [34]. The mode of failure showed a weak correlation with fracture strength, and no statistically significant difference was found between subgroups regarding failure mode. Nevertheless, the main failure mode was favorable in 75% of the mono-structured occlusal veneers and was favorable in 65.6% of the bi-structured occlusal veneers Consequently, it was determined that SFRC did not improve the mode of failure, which is consistent with the findings of Rocca et al., [35] as the randomly oriented fibers of the SFRC did not have an influence on the crack propagation front. However, this was different than the findings of Fráter et al. [27] & Magne et al. [10] who concluded that adding a SFRC core improves the survival rate and mode of failure of direct restorations, with fibers acting as cracks stoppers and stress absorbers [10, 27]. This may have been attributed to the thickness of the SFRC core, as in their studies the SFRC core thickness was 2.5–3 mm and 1–2.5 mm respectively, while the thickness of the SFRC core in this study was 1 mm [10, 27]. 

## Conclusions

This study tests the fracture strength of direct occlusal veneers with a SFRC core using a universal testing machine. Single loading did show that the fracture strength was high enough to withstand the maximum masticatory forces on premolars. However, external factors in the oral cavity still need to be taken into account that can affect the materials’ properties, including wear, cyclic loading, aging, temperature changes, and water sorption. Accordingly, further research studying these external factors is advised to mimic the clinical situation before shifting to in-vivo studies.

Under the limitations of this study, it can be concluded that:

- Fracture strength of direct occlusal veneers is greatly enhanced when supported by a SFRC core.
- High viscosity SFRC (EverX Posterior) and low viscosity SFRC (EverX Flow) provide similar support as cores for direct occlusal veneers.
- Injectable composite resin (G-aenial injectable) exhibits greater fracture strength than packable composite resin (G-aenial A’CHORD) when used as the veneering material for direct occlusal veneers.

## Data Availability

No datasets were generated or analysed during the current study.
